# Predictors of iatrogenic atrial septal defects: analysis of fibrotic atrial cardiomyopathy, valvular disease, and transseptal sheath size

**DOI:** 10.1007/s10840-025-02065-0

**Published:** 2025-05-19

**Authors:** Emanuel Heil, Jin-Hong Gerds-Li, Matthias Bock, Frank Heinzel, Gerhard Hindricks, Felix Hohendanner

**Affiliations:** 1 Deutsches Herzzentrum der Charité (DHZC), Berlin, Germany; 2 DZHK (German Centre for Cardiovascular Research), Partner Site Berlin, Berlin, Germany; 3 Städtisches Klinikum Dresden, Dresden, Germany

**Keywords:** Atrial cardiomyopathy, Atrial fibrillation, Ablation, Atrial septal defect, Transseptal puncture

## Abstract

**Background:**

Transseptal puncture (TSP) for left atrial access is routinely used during various cardiac interventions, including ablation for atrial tachyarrhythmia. However, in selected patients, subsequent iatrogenic atrial septal defects (iASD) persist. This study determines whether fibrotic atrial cardiomyopathy (FACM) or mitral valve regurgitation (MR) are predictors of persistent iASD development post-TSP.

**Methods:**

We analyzed data from patients undergoing radiofrequency ablation with high-density electroanatomical mapping for recurrent atrial tachyarrhythmias after a primary pulmonary vein isolation using either cryo or RF technologies. Patients were categorized based on transesophageal echocardiography findings: (1) competent atrial septum (cAS) (2), iASD, or (3) a patent foramen ovale (PFO). Differences in FACM and MR were assessed across these groups.

**Results:**

Of 149 patients (age 67.7 ± 9.7 years), 125 (83.9%) had cAS, 8 (5.4%) iASD, and 16 (10.7%) PFO. No significant differences were observed in age (*p* = 0.932), BMI (*p* = 0.612), or LVEF (*p* = 0.581). The TSP sheath size was not associated with iASD occurrence (*p* = 0.857). Common surrogates of FACM, i.e., LAVI (*p* = 0.114), LA area (*p* = 0.156), mean left atrial pressure (LAP; *p* = 0.459), or total low-voltage area burden (*p* = 0.058) did not differ significantly among groups. MR was not linked to increased LAP (at first (*p* = 0.290) and second procedure (*p* = 0.212)) or a higher incidence of iASD (at first (*p* = 0.155) and second procedure (*p* = 0.917)). Mean LAP did not correlate with LA size (*p* = 0.471) or low-voltage extent (*p* = 0.084).

**Conclusion:**

Our findings underscore that iASDs post-TSP for left atrial ablation are uncommon and unrelated to TSP sheath size, FACM, or MR, further minimizing concerns for routine interventions in patients with more advanced arrhythmia substrate or valvular disease.

**Graphical abstract:**

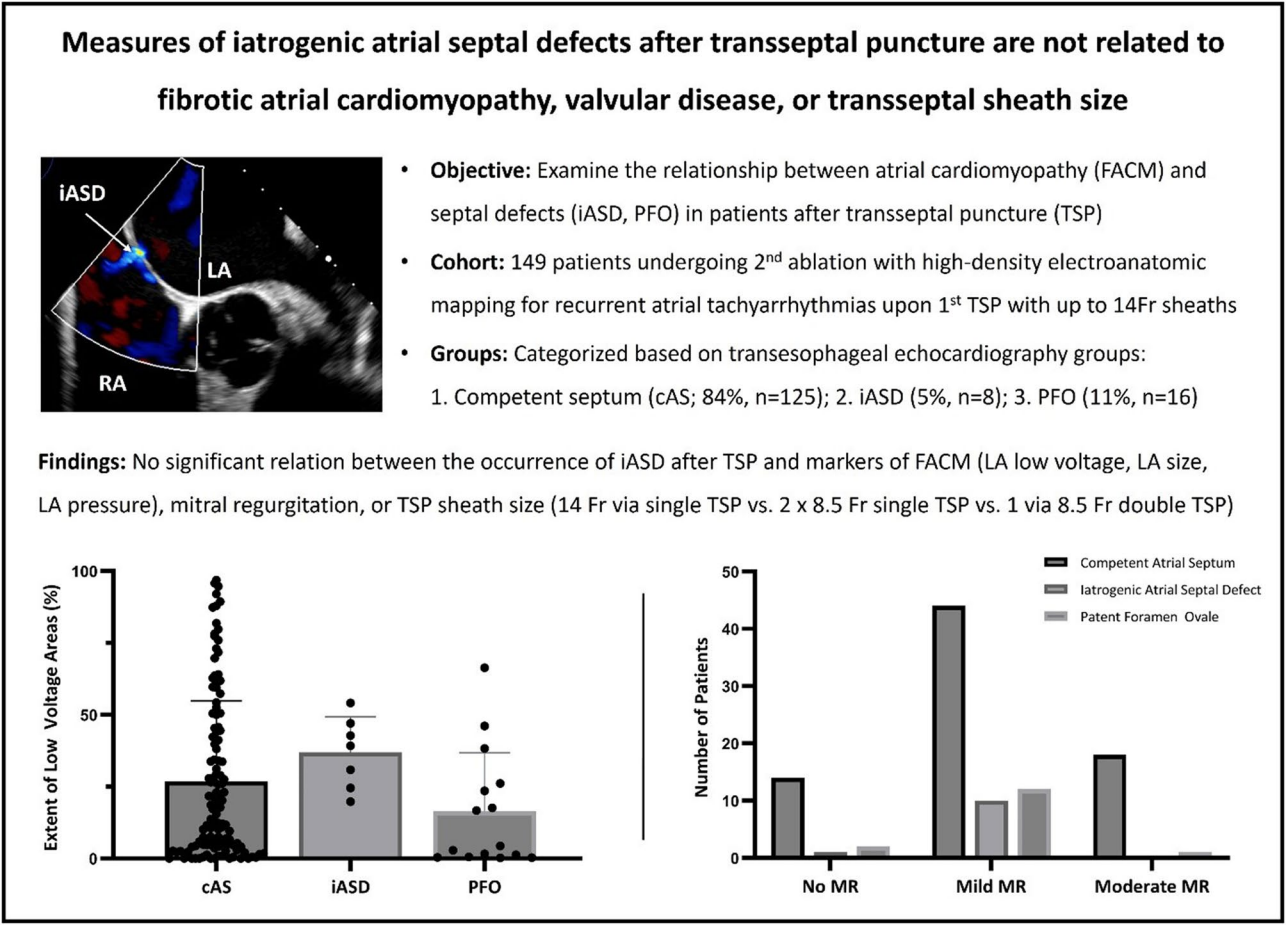

## Introduction

Transseptal puncture (TSP) has become an indispensable technique in modern interventional cardiology. Initially introduced by John Ross Jr. and Eugene Braunwald in 1959 to appropriately select patients for cardiac valve surgery, left atrial access is used today to treat structural heart diseases, atrial rhythm disorders, and left atrial appendage (LAA) occlusion [1, 2].

Utilization of real-time imaging techniques like transesophageal echocardiography (TEE) and intracardiac echocardiography (ICE) significantly improved the safety and efficacy of TSP [3] even though pure fluoroscopy-guided punctures remain the standard in a large number of centers. The puncture site is expected to heal within three to six months [4–6]. However, in up to 10% of patients, an iatrogenic atrial septal defect (iASD) persists upon TSP. This can potentially lead to complications such as embolic stroke and interatrial shunting. The indication for interventional closure in this set of patients remains elusive, and a close follow-up in patients with iASD is sometimes recommended [7, 8].

Predicting the persistence of iASD remains difficult. However, fibrotic atrial cardiomyopathy is potentially associated with impaired healing, and the impact of mitral valve regurgitation as a driver of pulmonary hypertension remains unclear. While prior studies have associated iASDs with procedural outcomes, the role of underlying atrial cardiomyopathy and valve disease in iASD persistence remains unclear.

This study, therefore, explores whether atrial cardiomyopathy and valvular heart disease represent significant risk factors for the persistence of iASD following TSP.

## Methods

### Study design

For this retrospective study, we included patients who underwent primary pulmonary vein isolation (PVI) using either cryoballoon or radiofrequency ablation, followed by secondary radiofrequency ablation with high-density 3D electroanatomical voltage mapping (Carto Version 7, Johnson & Johnson) and periprocedural echocardiography data between 2018 and 2024.

All patients were included regardless of sex, age, or whether the primary procedure was performed at our center or externally, provided they underwent preprocedural transesophageal echocardiography (TEE) before both the primary (1st) and secondary (2nd) ablation procedures. Eligibility requires venous blood samples and electrocardiograms (ECGs) collected at admission and preprocedural screening for left atrial thrombus and atrial septal defects.

Exclusion criteria included the absence of preprocedural TEE before the secondary procedure.

The cohort was divided into three groups based on TEE findings before the 2nd ablation:

- Patients with competent atrial septum (cAS).
- Patients with iatrogenic atrial septal defect (iASD).
- Patients with patent foramen ovale (PFO).

During TEE, atrial septal defects were evaluated for left-to-right shunting using color Doppler in a short-axis view of the aortic valve, with both the atria and the atrial septum visible. An atrial septal defect was classified as an iASD if it was newly developed after the first procedure (based on site-by-site TOE comparisons) and was centrally located in the atrial septum, outside the suspected fossa ovalis. A defect was classified as a PFO if it was located within the fossa ovalis, exhibited a flap-like movement, and appeared as a tunnel-like structure due to incomplete fusion of the septum primum and septum secundum. The size of an atrial septal defect was measured with the caliper tool.

All patients received at least one transthoracic echocardiography as part of standard care. During the ablation procedure, left atrial pressures (LAP) were recorded immediately upon TSP. Patients were lightly sedated (spontaneous breathing sedation) during pressure measurements using propofol. Data collected for each group included demographic information, left atrial volume index (LAVI), LAP, total left atrial wall area, and the extent of low-voltage regions (LVA, voltage < 0.5 mV) using 3D electroanatomical mapping. Mitral valve regurgitation (MR) severity was assessed during primary and secondary interventions. Data was analyzed within the framework of the local ethics committee approval EA1_020_25.

### Statistical analysis

Continuous variables are presented as mean ± standard deviation, while ordinal and non-normally distributed variables are expressed as median with interquartile range (IQR). Categorical data are reported as percentages. Equality of variances was evaluated using Levene’s and F tests. Differences among interval-scaled subgroups were assessed using t-tests, Mann–Whitney U tests, Kruskal–Wallis or Jonckheere-Terpstra tests as appropriate. Bonferroni correction was applied to adjust for multiple comparisons. Chi-Square and Fisher’s Exact tests were used to detect deviations for nominal and ordinal scaled variables. If appropriate, a Monte Carlo Simulation was utilized to correct for small sample size or distribution. Correlation analyses were conducted based on the data distribution, adhering to Cohen’s guidelines for interpreting effect sizes: small (*r* = 0.10), medium (*r* = 0.30), and large (*r* = 0.50). A p-value of ≤ 0.05 was considered statistically significant.

All statistical analyses were conducted using SPSS Statistics for Windows (version 29.0; IBM Corp., Armonk, NY), GraphPad Prism (version 10.4.1, GraphPad Software Inc., Boston, MA), and Microsoft 365 Excel Apps for Enterprise (version 2410; Microsoft, Redmond, WA).

## Results

**Fig. 1:**
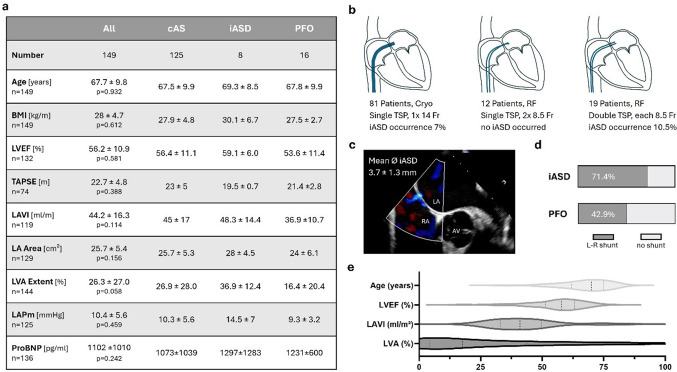
Study cohort. **a** Measurements during the 2nd procedure and significance levels for variations between groups. BMI Body Mass Index, LVEF Left Ventricular Ejection Fraction, TAPSE Tricuspid Annular Plane Systolic Excursion, LAVI Left Atrial Volume Index, LA Area Left Atrial Planimetric Area, LVA Extent Low Voltage Area Extent, LAP Mean Left Atrial Pressure. **b** Transseptal left atrial access during the first procedure and the associated incidence of iASD **c** Example TEE of an iatrogenic atrial septal defect. LA Left Atrium RA Right Atrium AV Aortic Valve. **d** Distribution of patients with left-to-right shunt and iatrogenic atrial septal defects or patent foramen ovale. **e** Age, LVEF, LAVI, and LVA extent in the cohort during the 2nd procedure

### Patient cohort and general findings

Key data from the cohort obtained during the second procedure are presented in Fig. 1a. Of these, 125 patients (83.9%) had a competent atrial septum (cAS), 8 (5.4%) presented with an iatrogenic atrial septal defect (iASD), and 16 (10.7%) had a patent foramen ovale (PFO). There were no significant differences across all groups or in pairwise group comparisons with respect to age (*p* = 0.932), body mass index (BMI; *p* = 0.612), left ventricular ejection fraction (LVEF; *p* = 0.581) or levels of proBNP (*p* = 0.242). The mean iASD diameter was 3.7 ± 1.3 mm, with left-to-right shunting observed in 71% of iASD cases and 43% of PFO cases (Fig. 1d).

### First procedure

All patients underwent pulmonary vein isolation as their initial procedure. Among the 149 patients, cryoballoon ablation using a single 14 Fr transseptal sheath was performed in 88 cases (59%). In 42 cases (28%), radiofrequency (RF) energy was delivered via two 8.5 Fr transseptal sheaths (13 single punctures, 23 double punctures, and 6 cases with unknown puncture details). In two additional cases (1%), a single transseptal sheath was used for ablation with an alternative RF catheter system. In 17 cases (11%), the energy source and the transseptal technique were unspecified.

Among the 16 patients with PFO, left atrial access for cryoballoon ablation with a 14 Fr sheath was achieved via the native opening in three cases. In nine cases, a transseptal puncture was required (5 single, 4 double), while in four patients, this information was unavailable. After excluding all PFO cases, iASD was observed in 6 of 81 cryoballoon ablation cases (7%) and 2 of 37 RF ablation cases (5%) (*p* = 0.857) (see Fig. 1b). In the RF group, both cases of iASD occurred in patients who had undergone double transseptal punctures with only a single sheath crossing each. In contrast, no iASDs were observed in patients where two sheaths were advanced through a single transseptal puncture. However, when comparing patients with a single transseptal puncture (cryoballoon + corresponding RF subgroup) to those with double transseptal punctures for RF ablation, no statistically significant difference in iASD occurrence was found (*p* = 0.512). No iASD was observed in patients with an unspecified energy source.

During the first procedure, the cohort demonstrated a mean left atrial pressure (LAP) of 9.5 ± 4.1 mmHg and a left atrial area (LA Area) of 26.1 ± 5.7 cm². Mitral regurgitation (MR) severity was distributed as follows: 81% of patients had none or mild MR, while 19% had moderate MR. No significant correlations were observed between moderate MR and LAP (*p* = 0.290). 14% of patients suffered (14 of 99 patients, no report available in 50 patients) from moderate to severe tricuspid valve regurgitation.

### Second procedure

The second procedure occurred an average of 37.6 ± 41.9 (cAS 35.9 ± 40.6, iASD 32 ± 41.6, PFO 53 ± 51) months after the first, with an average number of previous ablation attempts for atrial fibrillation of 1.3 ± 0.6. LVA was obtained from 3D electroanatomic maps during the 2nd procedure, which was 26 ± 27%. Low voltage preferentially occurred at the anterior wall compared to the posterior (28 ± 30% vs. 20 ± 27%, *p* < 0.001). Patients exhibited a mean LAP of 10.4 ± 5.5 mmHg (not significantly different from the first; *p* = 0.067) and an LA Area of 25.7 ± 5.3 cm². MR severity remained stable, with 70% showing none or mild MR and 19% moderate MR (no report in 16 patients). 8% (12 out of 144, no report in 5 patients) had documented moderate to severe tricuspid valve regurgitation.

### Fibrotic atrial cardiomyopathy

**Fig. 2:**
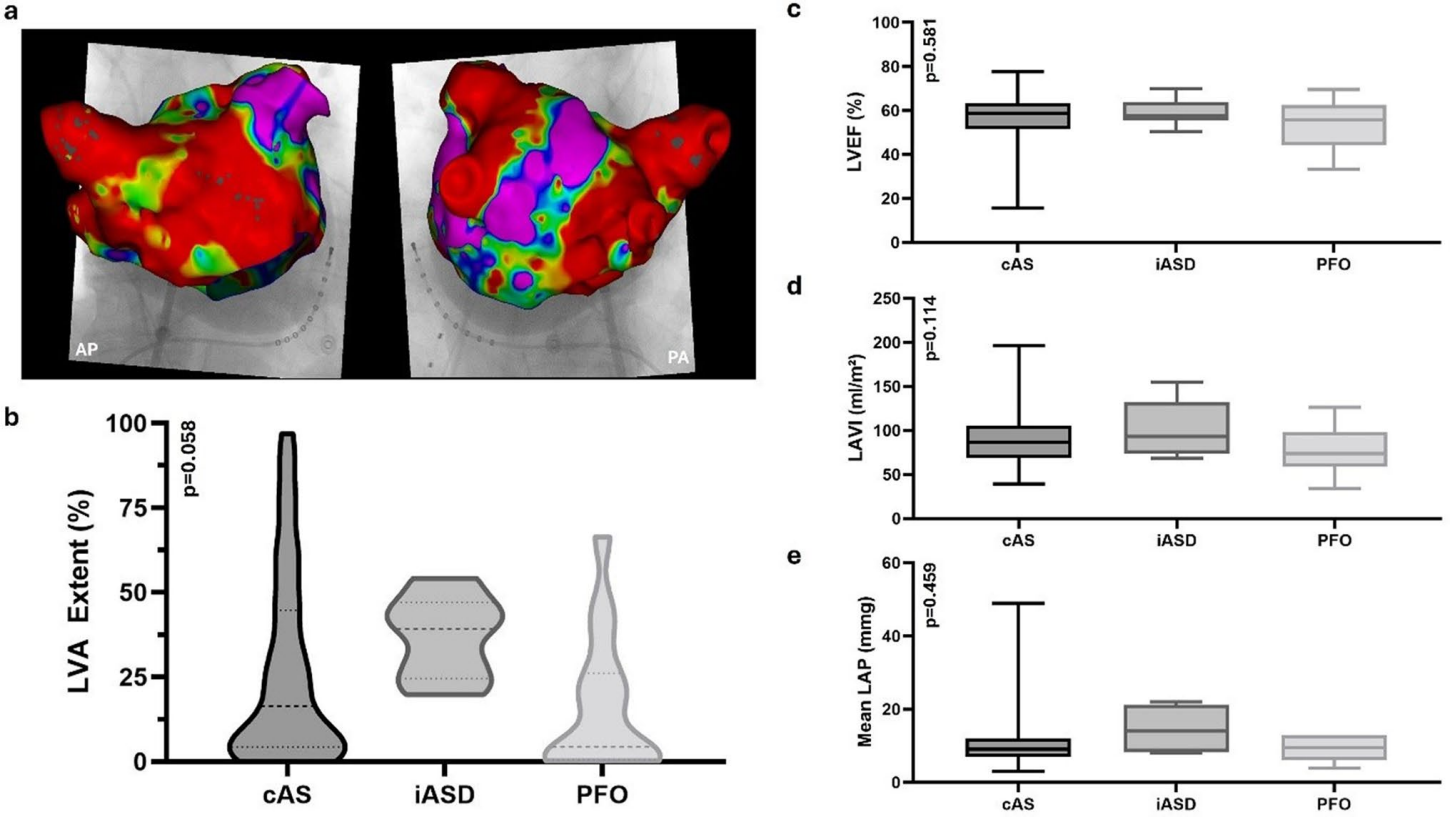
Iatrogenic Atrial Septal Defects in Fibrotic Atrial Cardiomyopathy. **a** CARTO Map example as used for determining LA low-voltage. **b** Distribution of low-voltage area burden in patients with competent atrial septum (cAS), iatrogenic atrial septal defects (iASD), and patent foramen ovale (PFO). **c** Left Ventricular Ejection Fraction across patients with cAS, iASD, and PFO. **d** Left Atrial Volume Index across patients with cAS, iASD, and PFO. **e** Mean Left Atrial Pressure across patients with cAS, iASD, and PFO

To evaluate the role of FACM in the development of iASDs, key surrogate markers were analyzed. No significant differences were observed in left atrial volume index (LAVI; *p* = 0.114), left atrial area (LA area; *p* = 0.156), mean left atrial pressure (LAPm; *p* = 0.459), or total LVA burden (*p* = 0.058) among patients with cAS, iASD, or PFO, either overall or in pairwise comparisons. Furthermore, LAPm in this cohort demonstrated no correlation with LA Area (*p* = 0.471) or LVA extent (*p* = 0.084). Regional analysis revealed no significant differences in anterior (*p* = 0.264) or posterior (*p* = 0.253) wall LVA burden among the groups (Fig. 2).

### Assessment for mitral valve regurgitation

**Fig. 3:**
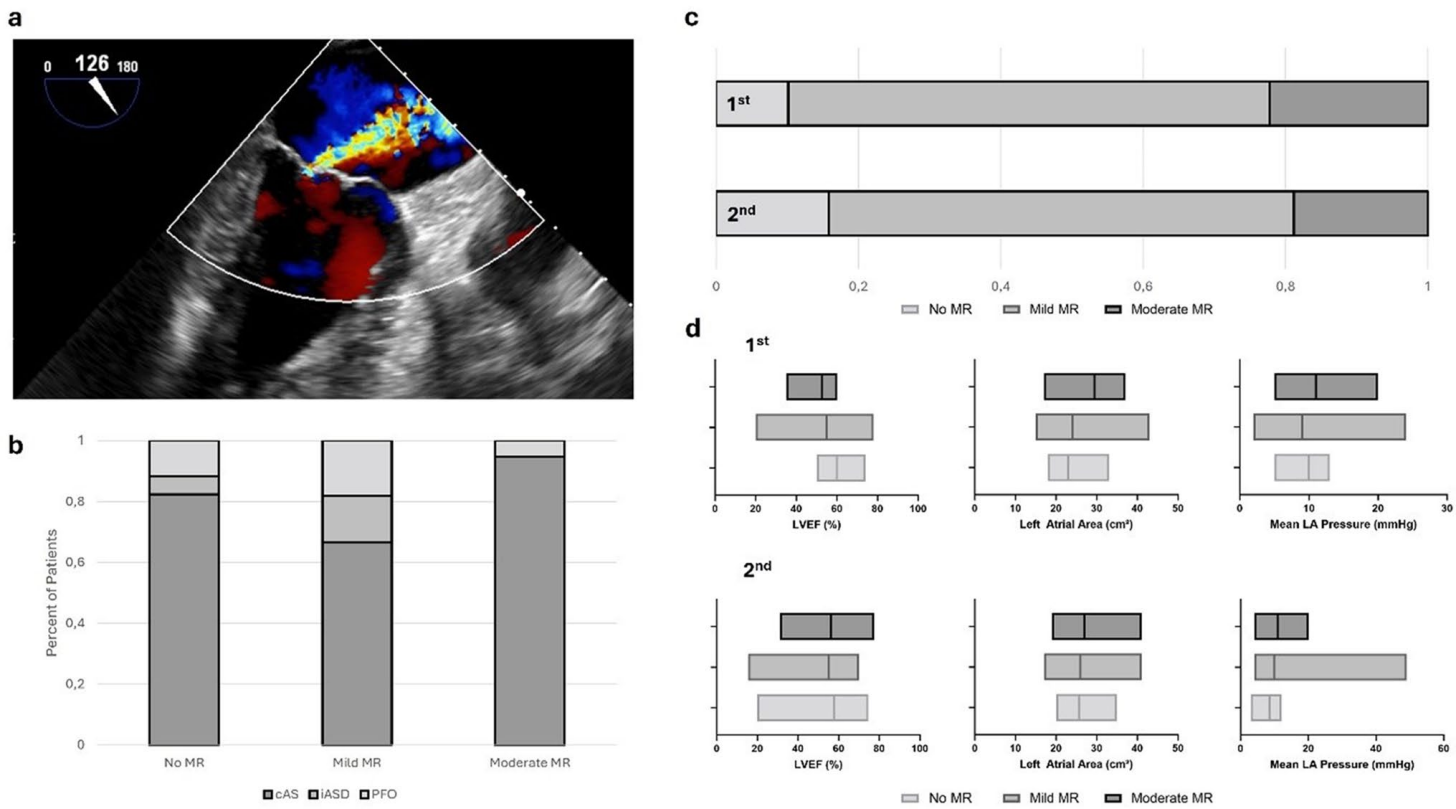
Iatrogenic Atrial Septal Defects in Mitral Valve Regurgitation. **a** TEE findings of moderate mitral valve regurgitation. **b** Distribution of Left Ventricular Ejection Fraction (LVEF), Left Atrial Planimetric Area (Left Atrial Area), and Mean Left Atrial Pressure at the first and second procedures across varying degrees of mitral valve regurgitation. **c** Distribution of septal competence across different levels of mitral valve insufficiency. **d** MR levels for competent atrial septum (cAS) and iatrogenic atrial septal defects (iASD) at first and second procedure

The severity of mitral valve regurgitation (MR) was assessed in a subgroup of 102 patients (66 male, 36 female) across both interventions, with an average interval of 31 ± 36 months. Left ventricular ejection fraction (LVEF) was preserved across procedures (first: 55 ± 10%, *n* = 84; second: 55 ± 11%, *n* = 92). The left atrium showed slight enlargement, with an LA area of 26 ± 5.8 cm² (first: *n* = 90) and 27 ± 5.6 cm² (second: *n* = 96) (see Fig. 3b).

At the first intervention, 83 patients (82%) exhibited no or mild MR, and 19 patients (18%) had moderate MR. No cases of severe MR were observed. By the second intervention, MR severity distribution remained stable, with 78% of patients showing no or mild MR and 22% having moderate MR (see Fig. 3c).

Comparative analysis (Fig. 3b) revealed no significant differences in key clinical parameters BMI (first: *p* = 0.851, second: *p* = 0.818), LVEF (first: *p* = 0.045, second: *p* = 0.480), or LA area (first: *p* = 0.043, second: *p* = 0.696). Additionally, mean left atrial pressure (LAP) at both interventions showed no relevant deviations (first: *p* = 0.500, second: *p* = 0.166), and MR severity demonstrated no significant correlation with LAP (first: τ = 0.146, *p* = 0.290; second: τ = 0.114, *p* = 0.212). We found no significant relation between MR level and the atrial septum competence at first (*p* = 0.155, φ = 0.264) and second procedure (*p* = 0.917, φ = 0.082). Notably, all patients with the occurrence of iatrogenic septal defects had no or mild MR at the first intervention (see Fig. 3d).

## Discussion

This study, limited by its retrospective design and relatively small sample size, suggests no significant association between measures of atrial cardiomyopathy, mitral regurgitation, and the occurrence of iASD.

Traditionally, LAP and the left atrial LVA burden have been associated with tissue dysfunction and arrhythmia recurrence. From a pathological perspective, wound healing may be impaired in patients with fibrotic atrial cardiomyopathy due to shared biological pathways. Similar to scar formation in the skin, cardiac wound healing begins with the deposition of collagen type III, which is later replaced by collagen type I and crosslinked to stabilize the scar. This complex process depends on matrix metalloproteinases, proinflammatory cytokines, and growth factors, and these proteins also play a significant role in the progression of heart failure [9–12]. Our findings suggest that although atrial electrical dysfunction is evident in our patients, the intricate cell-cell interactions necessary for wound repair remain intact. However, our study design does not permit an evaluation of whether excessive scarring occurred or whether the number of previous transseptal crossings impacts wound healing. The role of LAP in the development of iatrogenic septal defects remains uncertain. While elevated atrial pressure is hypothesized to hinder transseptal wound healing, and patients with iatrogenic septal defects exhibited higher mean LAP and a greater incidence of left-to-right shunting, statistical analysis revealed no significant association. This lack of significance may be attributable to the study’s relatively small sample size and the only moderately elevated atrial pressures observed. Similar considerations must be taken for low voltage extent and left atrial size.

In a study of 293 patients, Yang et al. reported a correlation between iatrogenic septal defects (iASDs) and higher rates of atrial arrhythmia recurrence [13]. However, as the study did not include redo procedures with electroanatomical mapping during follow-up, the investigators were unable to ascertain whether these recurrences were attributable to pulmonary vein isolation failure or triggers originating from the atrial myocardium. Based on our findings, impaired septal healing is unlikely to be associated with incomplete pulmonary vein healing or frequent pulmonary vein reconnections. Nonetheless, it remains unclear whether the apparent absence of septal wound healing impairments in atrial cardiomyopathy can be generalized to the stability of atrial ablation lesions in the pulmonary veins and myocardium.

Mitral valve regurgitation was not associated with elevated LAP or an increased incidence of iASDs following transseptal puncture. Our data offers a real-world perspective, as patients with high-grade mitral regurgitation are typically considered unsuitable for rhythm control when significant valvular disease remains untreated. Interestingly, evidence from percutaneous mitral valve repair (PMVR) procedures suggests that septal defects may still occur, even after technically successful interventions. A meta-analysis of six studies identified residual mitral regurgitation > II° as a predictor of persistent iASDs. However, patients with such hemodynamically significant insufficiencies were not included in the ablation cohort analyzed in this study [14].

Persistent iatrogenic septal defects (iASDs) following transseptal puncture for left atrial ablation are relatively uncommon, with limited tools available to predict their occurrence or assess their clinical significance. Our study indicates that transseptal punctures in patients with a greater burden of atrial cardiomyopathy and elevated left atrial pressures do not lead to a higher incidence of iASD. This underscores the strong safety profile of ablation procedures, even in non-paroxysmal AF patients.

The primary recommendation for preventing iASD after a transseptal puncture is to utilize the smallest sheath possible that still fulfills the procedural requirements [4]. Although limited by sample size, our investigation did not reveal a higher incidence of iASD in patients undergoing a large sheath transseptal passage, whether dilated by one large sheath or two smaller ones, compared to those with double transseptal punctures with a single smaller sheath crossing each. Considering data from mitral valve interventions, where transseptal access is achieved using a 22 Fr sheath [15], using sheaths up to 14 Fr does not appear to surpass the critical threshold for iASD formation. Since crossing two 8.5 Fr sheaths was not associated with a higher incidence of iASD either, a relevant increase in risk is not expected with the emergence of pulsed-field ablation systems, which typically utilize 16 Fr transseptal sheaths. Nevertheless, future studies should evaluate whether this increase in sheath size is associated with a higher rate of iatrogenic septal defects.

## Conclusion

Our findings suggest that the occurrence of iatrogenic atrial septal defect after catheter ablation for atrial fibrillation is unrelated to the extent of fibrotic atrial cardiomyopathy or preexisting mitral valve regurgitation.

## Data Availability

The data underlying this article will be shared at a reasonable request by the corresponding author.
